# Analysis of the gut microbiota and fecal metabolites in people living with HIV

**DOI:** 10.1128/spectrum.00238-24

**Published:** 2024-09-18

**Authors:** Xuebin Tian, Yiwen Xie, Lifeng Yu, Peng Yao, Mingqing Dong, Changzhong Jin, Nanping Wu

**Affiliations:** 1 Cell Biology Research Platform, Jinan Microecological Biomedicine Shandong Laboratory, Jinan, Shandong, China; 2 State Key Laboratory for Diagnosis and Treatment of Infectious Diseases, National Clinical Research Center for Infectious Diseases, National Medical Center for Infectious Diseases, Collaborative Innovation Center for Diagnosis and Treatment of Infectious Diseases, The First Affiliated Hospital, Zhejiang University School of Medicine, Hangzhou, Zhejiang, China; 3 Department of Critical Care Medicine, The First Affiliated Hospital of Shandong First Medical University & Shandong Provincial Qianfoshan Hospital, Shandong medicine and Health Key Laboratory of Emergency Medicine, Shandong Institute of Anesthesia and Respiratory Critical Medicine, Jinan, Shandong, China; 4 Department of Infectious Disease, Zhejiang Qingchun Hospital, Hangzhou, Zhejiang, China; U.S. Food and Drug Administration, Jefferson, Arkansas, USA

**Keywords:** HIV/AIDS, microbiota, 16S rRNA sequencing, untargeted metabolomics, metabolites

## Abstract

The gut microbiome has a pivotal function in human immunodeficiency virus (HIV). However, the associated alterations in the gut microbiome-host interaction are unknown. Herein, we aimed to investigate the gut microbiota and fecal metabolites in people living with HIV (PLWH). We collected stool samples from 70 PLWH and 34 healthy controls (HCs) and carried out 16S rRNA gene sequencing and analyzed the metabolites using liquid chromatography-mass spectrometry. *Firmicutes*, *Proteobacteria*, *Actinobacteriota*, and *Bacteroidota* were the most abundant phyla in both groups. Among genera, the level of *Escherichia*-*Shigella* was upregulated significantly in the PLWH group, whereas in the HC group, *Bacteroides* spp. were upregulated. Prediction of microbial function indicated significant reductions in alanine, aspartate, glutamate, and histidine metabolism. Furthermore, a comparison of the fecal metabolites between the HC and PLWH groups identified 38 differentially abundant metabolites in four differentially enriched human metabolic pathways. According to Spearman correlation analysis, there are close relationships between four differentially abundant microbiota members and five differentially abundant fecal metabolites, which might influence particular human metabolic pathways. Our findings provide a basis for further experimental investigation of the contribution of the gut microbiota and its associated metabolites to HIV/AIDS, providing a novel perspective for the further study of HIV/AIDS.

**IMPORTANCE:**

Growing evidence demonstrates that the gut microbiota is associated with HIV. This study investigated changes in the gut microbiota and fecal metabolites in PLWH. We identified 38 differentially abundant metabolites in four differentially enriched human metabolic pathways. Moreover, close relationships were noted between the four differentially abundant microbiota members and five differentially abundant fecal metabolites, which might influence particular human metabolic pathways. Thus, to benefit PLWH, potential pathobionts could be reduced (e.g., g_*Enterococcus*); probiotics could be increased (e.g., g_*Faecalibacterium* and g_*Agathobacter*); or certain metabolites (e.g., N-acetyl-L-phenylalanine and trehalose) could be reduced by changes in diet or the use of nutritional supplements. Our results provide insights into the interaction between the gut microbiota and the host, identifying possible targets that might be beneficial for PLWH.

## INTRODUCTION

Human immunodeficiency virus (HIV) infection causes acquired immunodeficiency syndrome (AIDS), which is historically one of the most deadly infectious diseases to affect humans. After 40 years of the AIDS epidemic, it is estimated that HIV-1 has infected 84 million people worldwide, resulting in the death of approximately 40 million (1). In China, the prevalence and mortality of HIV/AIDS have increased over the long term (2). Recently, an increasing number of studies have focused on the gut microbiota, which has a vital function in sustaining intestinal homeostasis. There is growing concern over how to maintain a healthy gut microbiota (3). The symbiotic gut microbiota has been intensively investigated for its antimicrobial resistance, substance metabolism, and immune regulatory effects (3–6). Growing evidence demonstrates that the gut microbiota is associated with HIV (7–9). Dysbiosis caused by HIV was reported to be associated with a decrease in alpha diversity, an increase in *Gammaproteobacteria*, such as *Enterobacteriaceae*, and a reduction in populations of *Lachnospiraceae* and *Ruminococcaceae* (10). Additionally, the levels of several butyric acid-producing bacteria belonging to the *Lachnospiraceae* and *Ruminococcaceae* families were reduced in people living with HIV (PLWH) (9). However, analysis of the genome of the gut microbiome reveals only the microbial composition, without providing evidence of their actual activities. Moreover, the interactions among the gut microbiota, diet, and the host cannot be revealed using genomics alone (11).

This knowledge gap might be filled using metabolomics, which could provide complementary functional data for the microbiota and reveal the dynamics of the interaction between the host and its microbiota (12). Recent research has shown that metabolites originating from the gastrointestinal tract, predominantly produced or altered by gut microbes, play a vital role in regulating both innate and adaptive immune responses (13). Host cognitive behaviors, cardiovascular health, metabolic homeostasis, and immunoregulation are associated with metabolites produced or altered by the gut microbiota [such as tryptophan metabolites, secondary bile acids, or short-chain fatty acids (SCFAs)] (11, 14–16). Therefore, host disease-related metabolic pathways and biomarkers associated with disrupted biological processes could be identified using metabolomic analysis.

The little research carried out on the impact of HIV/AIDS on the gut microbiota mostly employed gene sequencing approaches. Xie et al. found that HIV infection significantly changed the oral and gut microbiota (7, 17). Meyer-Myklestad et al. reported that probiotic supplementation altered the gut microbiota of PLWH (18). However, there have been few reports on the use of metabolomics to study the impact of the microbiota on PLWH. Moreover, the complexity of the interactions between the host and the microbiota-host in PLWH is mostly unknown. Consequently, this study aimed to reveal the gut microbe-host interplay in PLWH, adopting a multi-omics approach involving genomic and metabolomic analyses. We further aimed to reveal risk factors and identify possible targets that might benefit PLWH.

## MATERIALS AND METHODS

### Design of the investigation and the enrollment of participants

We recruited a cohort of 104 participants, which included 70 PLWH and 34 healthy control (HC) individuals. The PLWH were diagnosed by the Disease Control and Prevention Center of Zhejiang Province and were recruited from the HIV clinic of the First Affiliated Hospital of Zhejiang University (Zhejiang, China) between November 2020 and December 2022 (Table S1). Table S1 also lists the clinical characteristics of all the participants. The two groups showed no significant discrepancies regarding the male:female ratio and age (*P* > 0.05). We excluded those who received probiotics and/or antibiotics within 4 weeks prior to enrollment.

### Sample collection and processing

The natural defecation method was used to collect the stool samples. To preclude contamination, urine was drained before defecation. Two grams of feces was placed in a sterile fecal sample collection tube using a sampling spoon. To inhibit bacterial growth, the tubes were placed on ice and then transferred to a −80 ℃ freezer within 120 min.

### Extraction of DNA

An Omega Mag-Bind Stool DNA kit (Omega Bio-Tek, Norcross, GA, USA) was employed to isolate total DNA from the microbiome of the stool samples. Agarose gel electrophoresis (1.2%) was employed to ascertain the quality of the isolated DNA. Paired-end sequencing of the DNA fragments was carried out on the Illumina platform (Illumina, San Diego, CA, USA), and the obtained sequencing data were retained in the FASTQ format.

### Metagenomic sequencing of 16S rRNA

The 16S rRNA V3–V4 variable region was amplified from the bacterial genome using PCR with forward 5′-ACTCCTACGGGAGGCAGCA-3′ and reverse 5′-GGACTACHVGGGTWTCTAAT-3′ primers. We constructed the high-throughput sequencing library employing an Illumina TruSeq Nano DNA LT library prep kit, followed by sequencing on the Illumina platform. The original sequence data were optimized employing the dada2 method in the Quantitative Insights into Microbial Ecology2 (QIIME2) software (v.2019.4) (19). The Greengenes database (http://ftp.microbio.me/greengenes_release/current/) was searched using default parameters to taxonomically allocate the sequences using QIIME2, and species annotation was carried out using the pretrained naive Bayes classifier (https://github.com/QIIME2/q2-feature-classifier) (20) (21).

### Sample preparation for metabolome profiling

A Vanquish ultrahigh-performance liquid chromatography system (Thermo Fisher Scientific, Waltham, MA, USA) was used to carry out the liquid chromatography (LC) analysis, employing an ACQUITY UPLC HSS T3 column (150 × 2.1 mm, 1.8 µm) (Waters, Milford, MA, USA). The temperature of the column was set to 40℃. We set the flow rate to 0.25 mL/min, and the injection volume was 2 µL. For the LC-electrospray ionization (ESI) (+)-mass spectrometry (MS) analysis, the mobile phases comprised (C) 0.1% formic acid in acetonitrile (vol/vol) and (D) 0.1% formic acid in water (vol/vol). The following gradient was used for separation: 0–1 min, 2% C; 1–9 min, 2%–50% C; 9–12 min, 50%–98% C; 12.0–13.5 min, 98% C; 13.5–14.0 min, 98%–2% C; and 1,420 min, 2% C. For LC-ESI (−)-MS analysis, the mobile phases comprised (A) acetonitrile and (B) 5-mM ammonium formate. The following gradient was used for separation: 0–1 min, 2% A; 1–9 min, 2%–50%A; 9–12 min, 50%–98% A; 12.0–13.5 min, 98% A; 13.5–14.0 min, 98%–2% A; and 14–17 min, 2% A (22).

A Q Exactive HF-X column (Thermo Fisher Scientific) including an ESI ion source was used to carry out MS detection of the metabolites. We employed simultaneous MS1 and MS/MS (full MS-ddMS2 mode, data-dependent MS/MS) acquisition using the following parameters: Sheath gas pressure = 30 arbitrary units (arb), auxiliary gas flow = 10 arb, spray voltage = 3.50 kV [ESI (+)] and −2.50 kV [ESI (−)], capillary temperature = 325℃, MS1 range = *m*/*z* 100–1000, MS1 resolving power = 60,000 full width at half maximum (FWHM), number of data-dependent scans per cycle = 8, MS/MS resolving power = 15,000 FWHM, normalized collision energy = 30%, and automatic dynamic exclusion time (23).

### Statistical and bioinformatic analyses

All statistical calculations were carried out using R (v.4.2.1) (24). The participants’ ages are shown as means ± standard deviations. To analyze categorical variables, we employed Fisher’s exact test. Differences in the microbiota between the two groups were analyzed with the Wilcoxon rank-sum test, and differences in the metabolites between the two groups were determined using two-tailed Student *t*-tests. Community richness was assessed using the Chao1 index, and diversity was assessed using the Shannon index (25). The “vegan” R package was employed to carry out dissimilarity tests among groups (permutational multivariate analysis of variance) conducted on the Euclidean distance (metabolites) and the Bray-Curtis distance (bacteria), with 10,000 permutations. To identify biomarkers, differences in the microbial communities were assessed using linear discriminant analysis effect size (LEfSe) (26). Kyoto Encyclopedia of Genes and Genomes (KEGG) Orthology functional prediction of microbial metabolism was carried out using Phylogenetic Investigation of Communities by Reconstruction of Unobserved States 2 (PICRUSt2) from the KEGG database. Correlations between the differentially abundant bacteria and the top 50 differentially abundant fecal metabolites were determined using Spearman correlation analysis based on their relative abundances. Statistical significance was considered to be indicated by *P* < 0.05. Accuracy mass (<30 ppm) and MS/MS data were used to identify the metabolites, according to matches in the following databases: KEGG (http://www.genome.jp/kegg/), mzcloud (https://www.mzcloud.org, LipidMaps (http://www.lipidmaps.org/), and HMDB (https://hmdb.ca/metabolites). We combined the positive and negative data, which were then imported into the R ropls package. Orthogonal partial least-squares discrimination analysis (OPLS-DA) was carried out to visualize the metabolic alterations between the HC and PLWH groups.

## RESULTS

### Gut microbiome profile of PLWH

**Fig 1:**
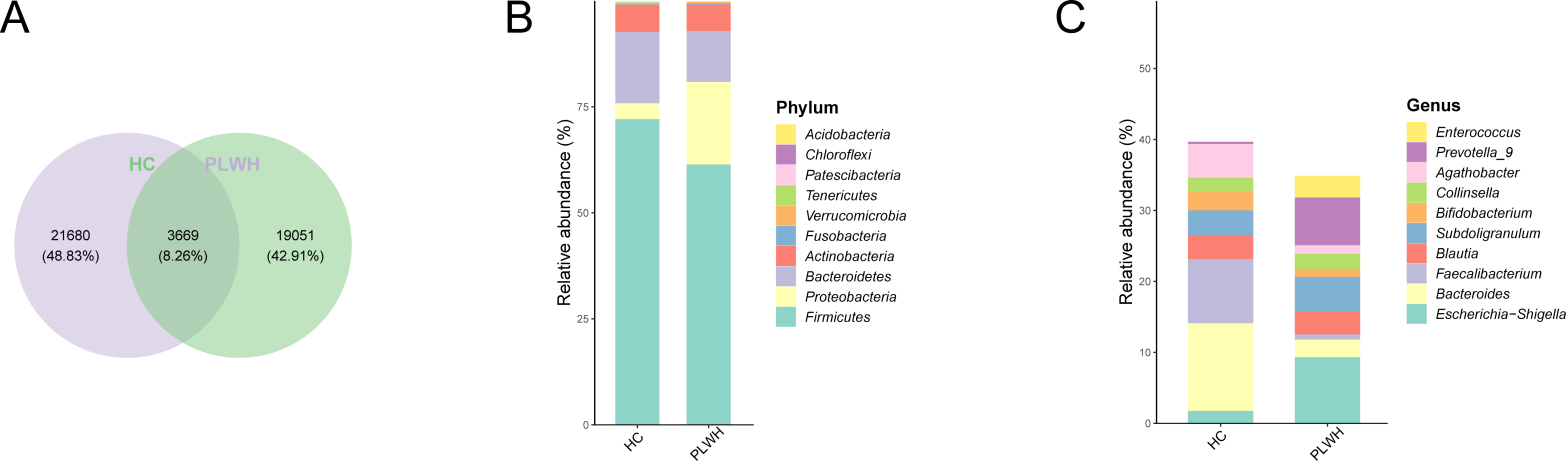
(**A**) Venn diagram for the PLWH and healthy control (HC) groups. (**B**) Histogram showing the community composition of the PLWH and HC groups at the level of phyla.(**C**) Histogram showing the community composition of the PLWH and HC groups at the level of genera.

Curve analysis of species accumulation (Specaccum) indicated that the slope flattened toward the right (Fig. S1A), suggesting that most of the species in the sample had been captured. The Venn diagram showed the PLWH and HC groups comprised a core of 3,669 amplicon sequence variants (ASVs). The PLWH group contained 19,051 unique ASVs, and the HC group contained 21,680 unique ASVs (Fig. 1A). At the level of phyla, *Firmicutes*, *Proteobacteria*, *Actinobacteriota*, and *Bacteroidota* comprised the major gut microbiota components (Fig. 1B). At the level of genera, in the PLWH group, *Escherichia*-*Shigella* was the most common genus, whereas in the HCs, it was *Bacteroides* (Fig. 1C).

### Analysis of microbial diversity

**Fig 2:**
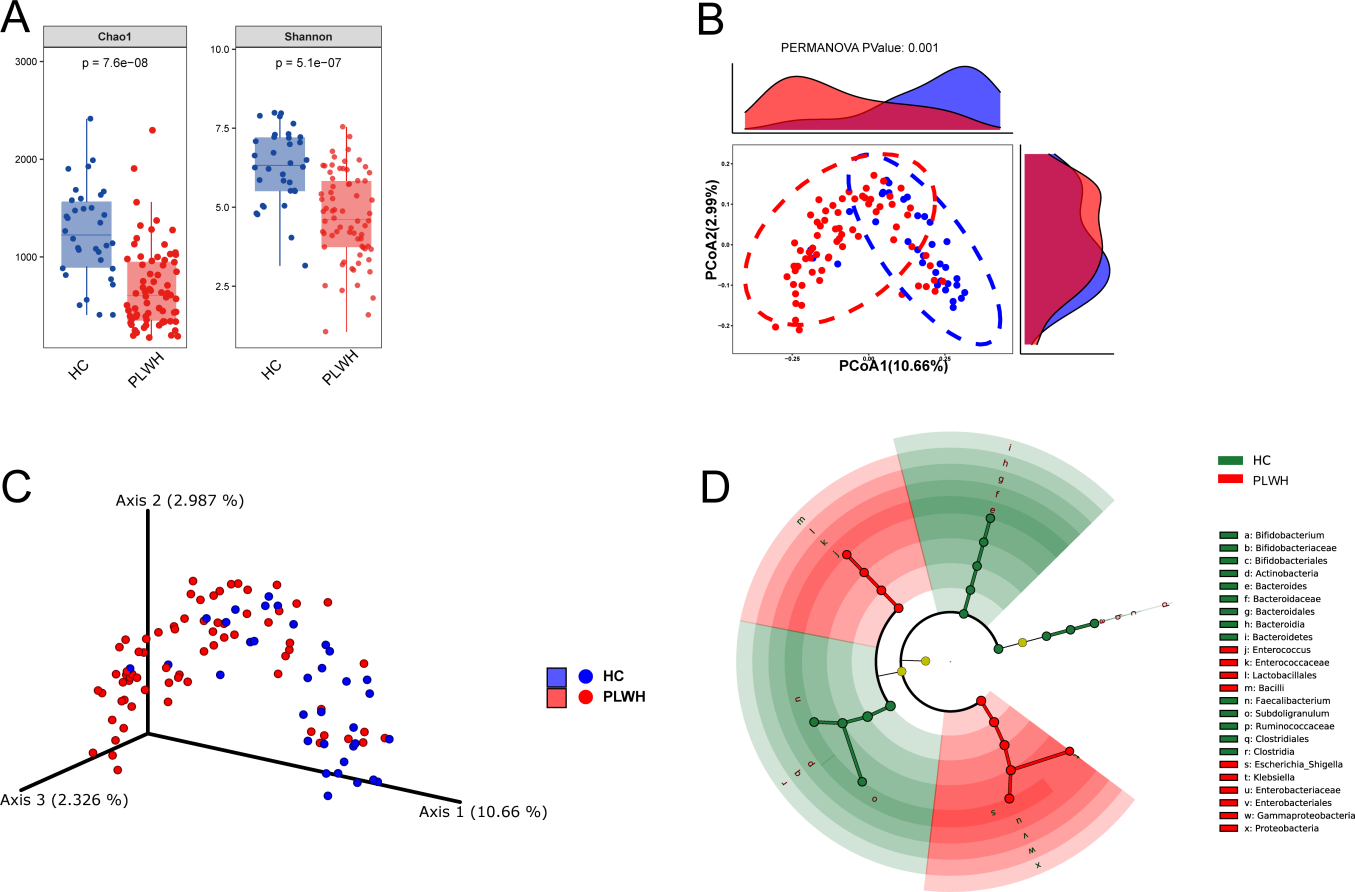
Microbial diversity and LEfSe analyses of the PLWH and HC groups. (**A**) Differences in species richness between the two groups are indicated by the Chao1 index. Differences in species evenness between the two groups are indicated by the Shannon index. (**B**) PCoA and (**C**) three-dimensional-PCoA scores indicating the beta diversity. (**D**) Diagram of taxonomic branches for significant bacterial species at a linear discriminant analysis threshold of 4.

According to the alpha diversity analysis, the species richness and species diversity of the HC group were higher compared with those of the PLWH group (*P* < 0.001) (Fig. 2A). Rank-abundance curves showed no significant differences (Fig. S1B). The data were then analyzed using principal-coordinate analysis (PCoA). Herein, PCo1 was 10.66%; PCo2 was 2.99%; and PCo3 was 2.33%, with the three coordinates representing 15.98% of the HC and PLWH groups. The HC and PLWH groups showed a marked difference in beta diversity between them (*R*^2^ = 0.046, *P* = 0.001), indicating that the two groups had differences in their microbiota composition. The PCoAs and three-dimensional PCoAs for the HC and PLWH data are displayed in Fig. 2B and C.

The linear discriminant analysis threshold was set to 4 for LEfSe exploration of the microbial species that showed significant differences between the HC and PLWH groups, which identified 24 enriched species (Fig. 2D). As shown in the chart, *Clostridia*, *Bacteroidaceae*, and *Bacteroides* displayed a somewhat high abundance in the HC group, while *Escherichia*-*Shigella* and *Klebsiella* displayed a somewhat high abundance in the PLWH group.

### Potential functions of the altered microbiota

**Fig 3:**
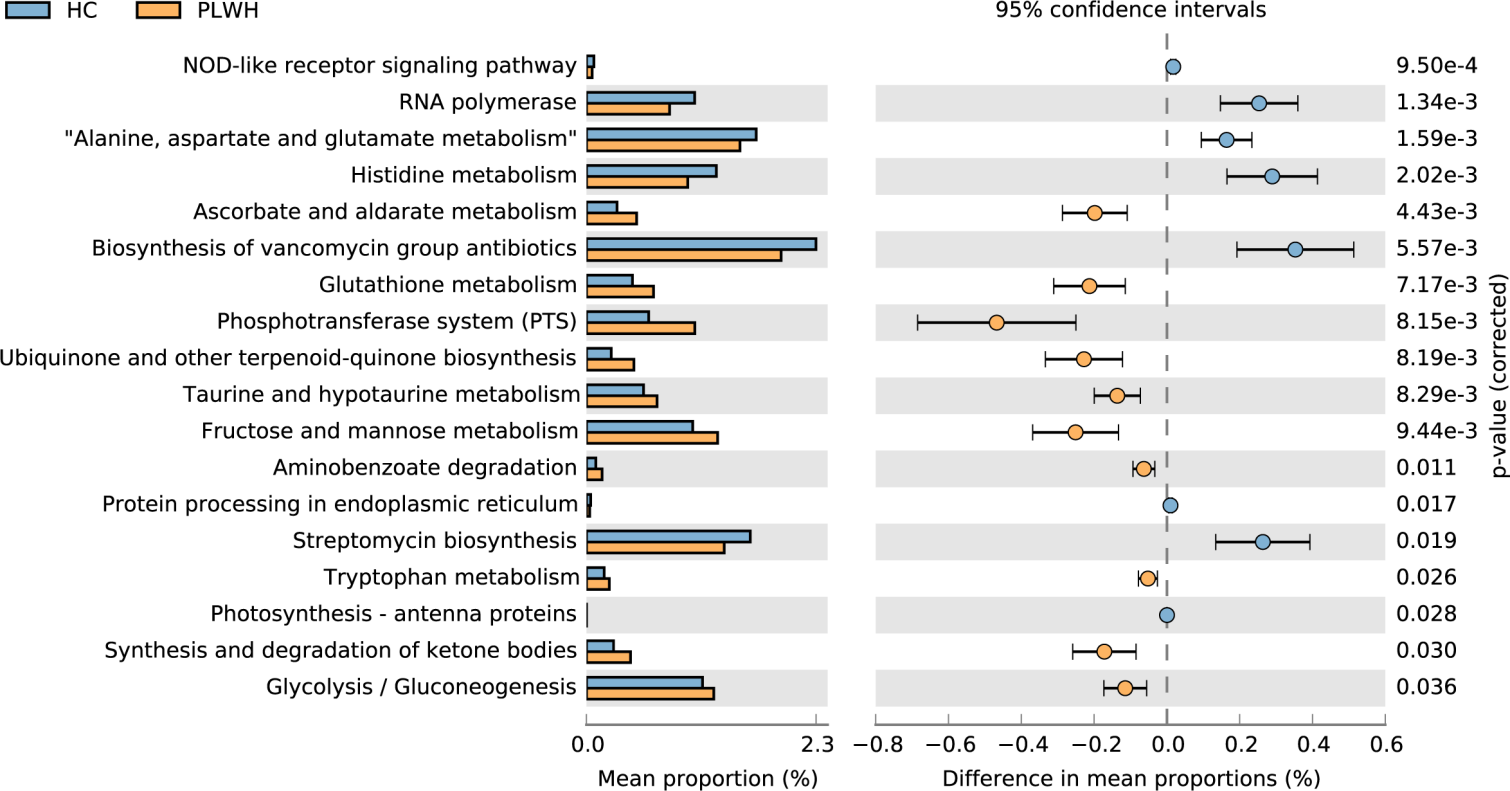
Predicted microbial functional analysis. Analysis using Phylogenetic Investigation of Communities by Reconstruction of Unobserved States 2, which identifies Kyoto Encyclopedia of Genes and Genomes Orthology level 3 pathways between the PLWH and HC groups.

The results of PICRUSt2 analysis suggested that glutathione metabolism, taurine and hypotaurine metabolism, fructose and mannose metabolism, and tryptophan metabolism had increased significantly in the PLWH group relative to that in the HC group, while the HC group showed significant increases in alanine, aspartate, glutamate, and histidine metabolism relative to that in the PLWH group (Fig. 3).

### LC-MS analysis of fecal metabolites

**Fig 4:**
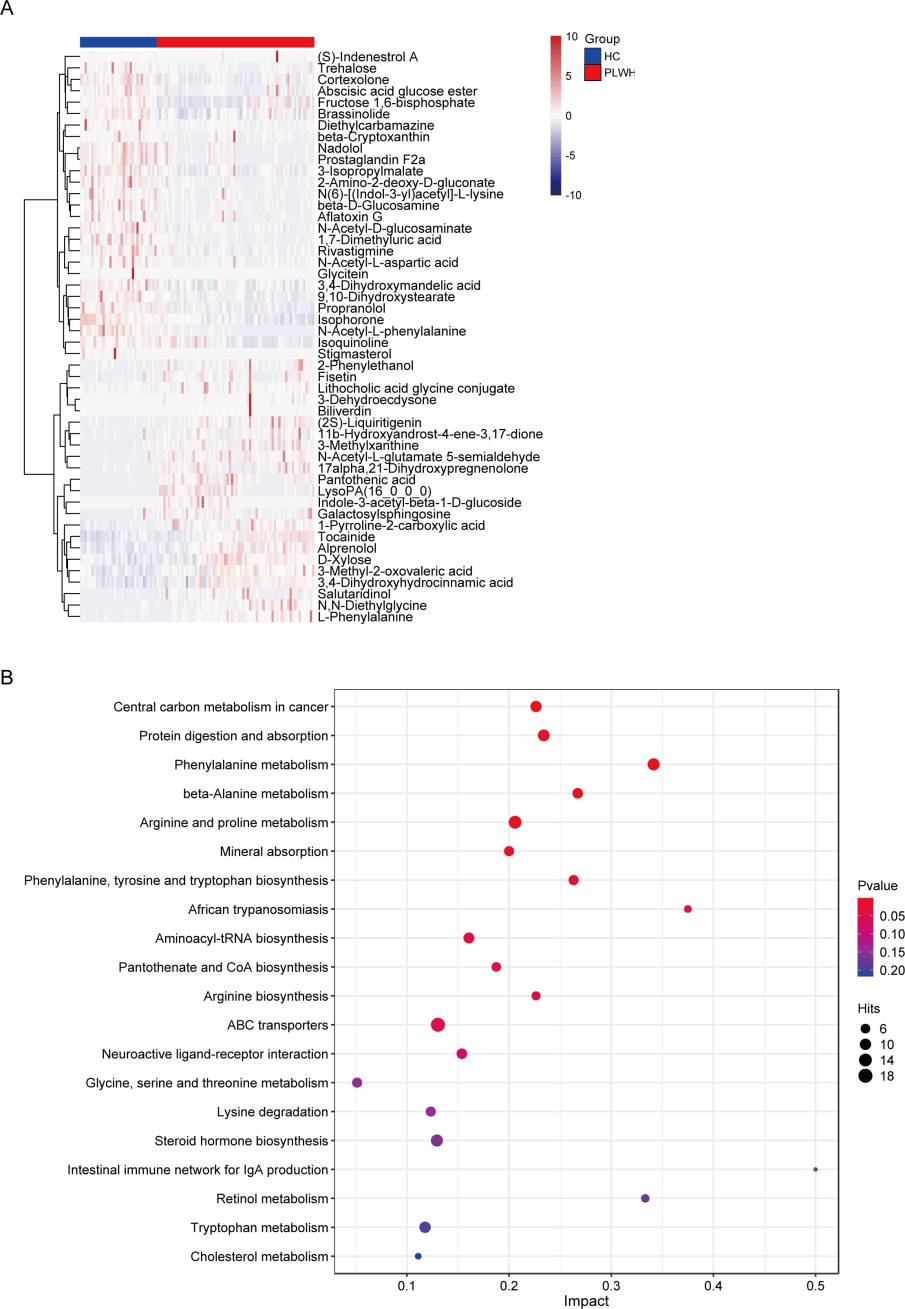
Fecal metabolite analysis using liquid chromatography-mass spectrometry. (**A**) The top 50 differentially abundant metabolites between the PLWH and HC groups shown as a heatmap. (**B**) The top 20 enriched KEGG metabolic pathways between the two groups shown as a bubble chart. The metabolic pathways are on the *y*-axis, and the rich factor (the number of significant differentially abundant metabolites/the total metabolites in the pathway) is on the *x*-axis. A larger rich factor indicates a greater degree of enrichment. Change in the color from blue to red indicates a decreasing *P* value; the bigger the dot, the higher the number metabolites enriched in the pathway.

To ascertain the effect of HIV infection on the fecal metabolites of PLWH, we carried out liquid chromatography-mass spectrometry (LC-MS) untargeted metabolomic analysis. OPLS-DA could effectively differentiate between the HC and PLWH groups (Fig. S2A). The presence of significant differentially abundant metabolites between the HC and PLWH groups was indicated by discrete points on the OPLS-DA S plot (Fig. S2B). Herein, we analyzed 829 fecal metabolites, among which 292 were differentially abundant. Figure 4 shows the top 50 differentially abundant metabolites. Metabolites showing a marked increase in the PLWH group were N-acetyl-L-glutamate 5-semialdehyde, 11b-hydroxyandrost-4-ene-3,17-dione, and 17alpha,21-dihydroxypregnenolone. Conversely, the HC group exhibited significantly elevated levels of N-acetyl-L-phenylalanine and trehalose.

### Predicted effects of the differentially abundant metabolites on human metabolism

The LC-MS-identified differentially abundant metabolites were annotated using the KEGG pathway mapper. A comparative investigation of the human metabolic profiles indicated marked alterations in central carbon metabolism and major metabolic pathways, such as protein digestion and absorption, and amino acid pathways (Fig. 4B). We decided to focus on 38 differentially abundant metabolites in four differentially enriched human metabolic pathways. According to their *P* values, phenylalanine metabolism; arginine and proline metabolism; the biosynthesis pathways of phenylalanine, tyrosine, and tryptophan, and arginine were characterized as significantly affected by HIV infection, with *P* values of 0.005, 0.007, 0.0256, and 0.0457, respectively (Fig. 4B).

### Correlations between LC-MS untargeted metabolomics and 16S rRNA gene sequencing

**Fig 5:**
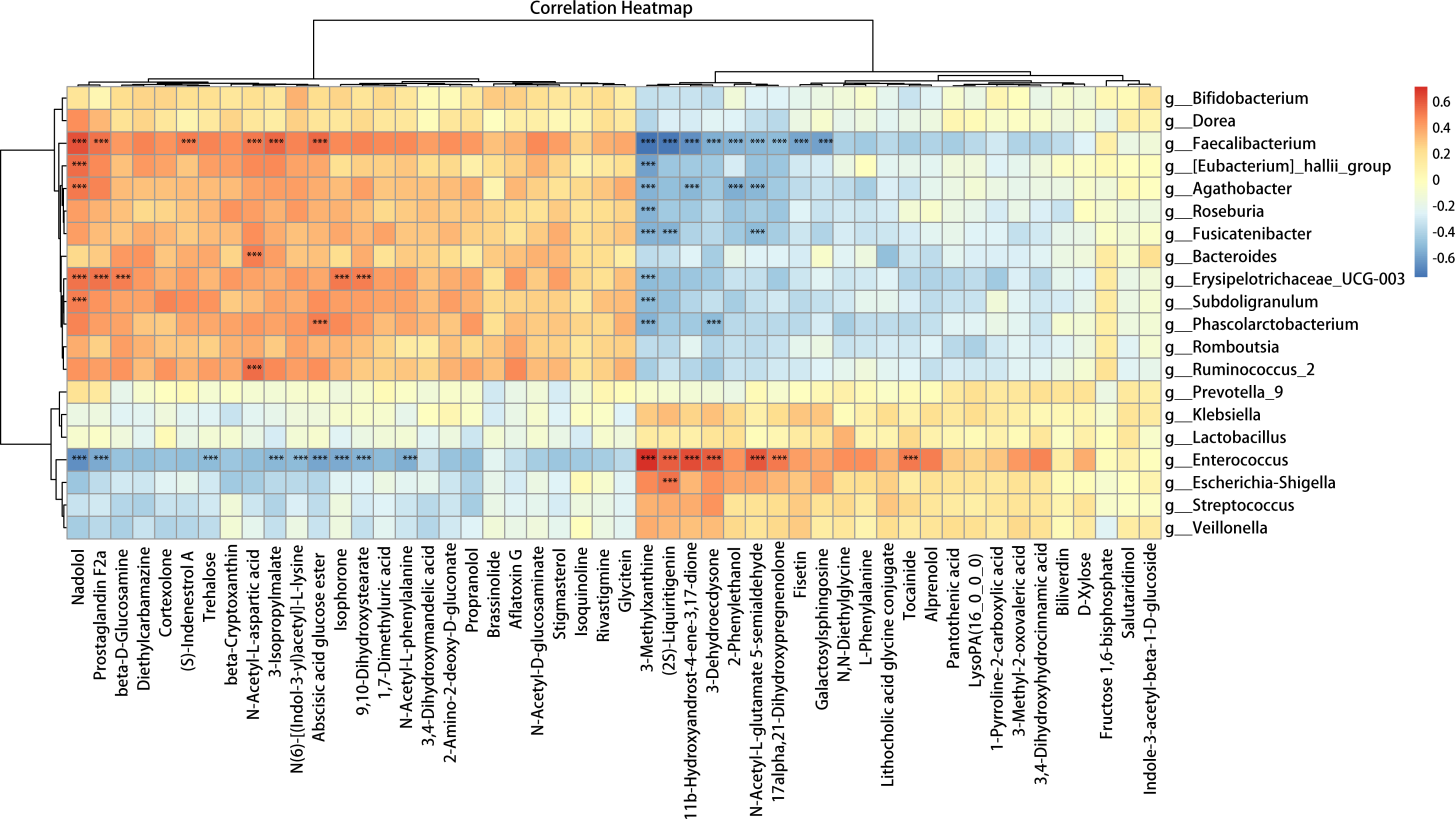
Correlation analysis of the top 50 differentially abundant metabolites and bacteria between the PLWH and HC groups shown as a heatmap. Associations between the differentially abundant bacteria and the top 50 differentially abundant fecal metabolites were analyzed using Spearman correlations based on their relative numbers.

We used Spearman correlation analysis to ascertain the associations among human metabolic pathways, fecal metabolites, and the gut microbiome, aiming to assess the associations between the differentially abundant bacteria and the top 50 differentially abundant fecal metabolites based on their relative levels (Fig. 5). The human KEGG pathway mapper could annotate 5 differentially abundant fecal metabolites among the top 50. We then tabulated the correlations among human metabolic pathways, altered bacteria, and the differentially abundant fecal metabolites (Table 1).

**TABLE 1:** Correlations among differential bacteria, differential fecal metabolites, and annotated human metabolic pathways^*a*^

Differential metabolite	Metabolite change	Differential microbiota member	Microbiota member change	Rho value	Correlation *P* value	Human pathway annotation(s)
N-Acetyl-L-glutamate 5-semialdehyde	↑	g_*Enterococcus* g_*Fusicatenibacter* g_*Agathobacter* g_*Faecalibacterium*	↑ ↓ ↓ ↓	0.601 −0.509 −0.525 0.521	1.521E-11 3.426E-08 1.021E-08 1.447E-08	Arginine biosynthesis
N-Acetyl-L-phenylalanine	↓	g_*Enterococcus*	↑	−0.545	2.208E-09	Phenylalanine metabolism
11b-Hydroxyandrost-4-ene-3,17-dione	↑	g_*Enterococcus* g_*Agathobacter* g_*Faecalibacterium*	↑ ↓ ↓	0.637 −0.515 −0.647	3.585E-13 2.179E-08 1.189E-13	Steroid hormone biosynthesis
17alpha,21-dihydroxypregnenolone	↑	g_*Enterococcus* g_*Faecalibacterium*	↑ ↓	0.514 −0.512	2.381E-08 2.808E-08	Steroid hormone biosynthesis
Trehalose	↓	g_*Enterococcus*	↑	−0.510	3.142E-08	ABC transporters

^*a*^ ↑ indicates upregulation and ↓ indicates downregulation.

**Fig 6:**
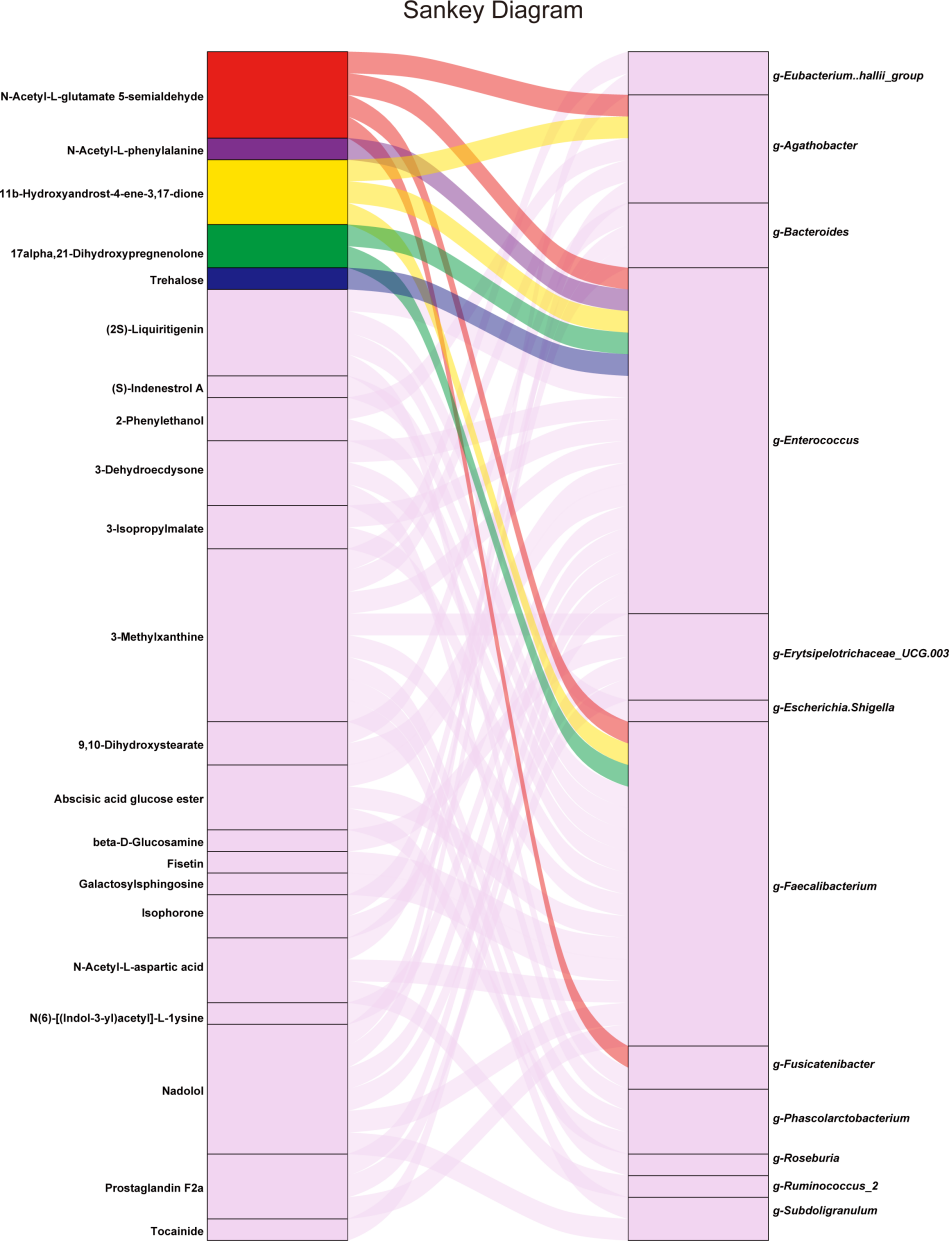
Correlations between differentially abundant bacteria and fecal metabolites. This Sankey diagram was constructed based on the following correlation analysis of the top 50 differentially abundant microbiota members and metabolites, we constructed this Sankey diagram based on a significance threshold of *P* < 0.05 to select the relevant correlations. The diagram highlights five differentially abundant metabolites (shown in color), which are annotated to human KEGG pathways, correlating with four differentially abundant microbes. The metabolites that were not identified as being associated with human KEGG pathways are shown in the background color. The overall size of the rectangle for each microbe or metabolite is representative of the number of correlations between them, providing a visual scale of interaction strength and relevance.

Four differentially abundant microbiota members were closely related to five differentially abundant fecal metabolites, which may influence particular human metabolic pathways. In particular, increased N-acetyl-L-glutamate 5-semialdehyde levels correlated with g_*Enterococcus*, g_*Fusicatenibacter*, g_*Agathobacter*, and g_*Faecalibacterium*, with a possible effect on arginine biosynthesis. The reduction in N-acetyl-L-phenylalanine levels correlated with g_*Enterococcus*, which might influence phenylalanine metabolism. 11b-Hydroxyandrost-4-ene-3,17-dione and 17alpha,21-dihydroxypregnenolone, which were significantly increased, and correlated with g_*Enterococcus* and g_*Faecalibacterium*, might influence steroid hormone biosynthesis. Moreover, reduced levels of trehalose correlated with g_*Enterococcus*, possibly influencing ABC transporters. We observed an association between g_*Enterococcus* and the greatest number of downregulated differentially abundant metabolites, followed by g_*Faecalibacterium* and g_*Agathobacter*. In particular, g_*Enterococcus* was associated with pathways such as phenylalanine metabolism, arginine biosynthesis, and steroid hormone biosynthesis. Thus, these species might carry out important and pleiotropic roles in the interaction between the microbiota and the host. Furthermore, the covariation between the differentially abundant metabolites and their associated gut microbes was illustrated using a Sankey diagram (Fig. 6).

**Fig 7:**
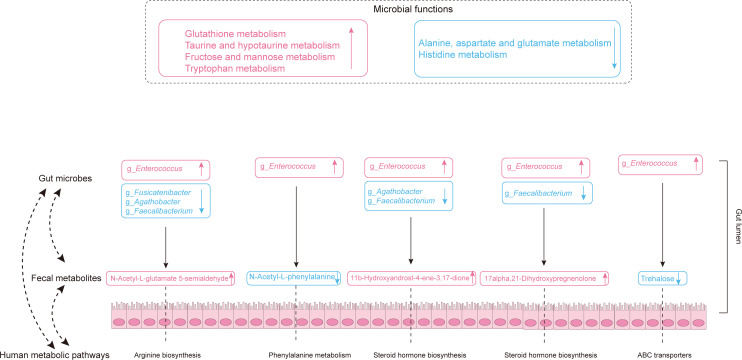
Diagram of the interactions between the gut microbiota and the host in the PLWH and HC groups. Text in pink represents augmented microbial functions, fecal metabolites, and potential pathobionts, while text in blue represents diminished microbial functions, fecal metabolites, and potential probiotics.

Together, these results suggest that HIV infection causes gut dysbiosis and could affect human metabolic pathways. Figure 7 shows a diagram of the interactions between the gut microbiota and the host resulting from HIV infection (Fig. 7).

## DISCUSSION

Herein, multi-omics analysis of 16S rRNA gene sequencing data and LC-MS untargeted metabolomics were used to analyze the differences in the gut microbiota composition between PLWH and HCs. The major findings were as follows: (i) the gut microbiota composition in the PLWH group differed significantly with that of the HC group; (ii) 38 differentially abundant metabolites in four differentially enriched human metabolic pathways were of interest; and (iii) four differentially abundant microbiota members were closely related to five differentially abundant fecal metabolites, according to Spearman correlation analysis, which may impact particular human metabolic pathways.

Herein, we revealed that in PLWH, the gut microbial composition was markedly changed, which agreed with the findings of previous studies (7, 27). Our findings showed that changes in the gut microbiota composition between the two groups led to significant differences in their functions, which were manifested by a significant decrease in histidine metabolism, and alanine, aspartate, and glutamate metabolism, in the PLWH group. The glycogenic amino acid alanine is present at a high percentage in many proteins. In the current study, alanine metabolism in the PLWH group decreased significantly relative to that in the HCs. This result might be associated with the decreased ability of the body to absorb and utilize nutrients because of HIV infection. Glutamate, aspartate, and histidine are precursors of many molecules. Moreover, glutamate, as a stimulant neurotransmitter, is important and highly active in the nervous system. Indeed, glutamate toxicity has a vital function in neurodegeneration and is related to HIV-associated neurocognitive diseases (28). Changes in the functional gene expression in the gut microbiome of PLWH and HCs require further investigation.

Herein, LC-MS untargeted metabolomics revealed that PLWH had 38 differentially abundant metabolites in 4 differentially enriched metabolic pathways. According to Spearman’s correlation analysis, four differentially abundant microbiota members showed close correlations with five differentially abundant fecal metabolites, which could exert particular effects on PLWH’s metabolic pathways. In particular, decreases in N-acetyl-L-phenylalanine and trehalose correlated with g_*Enterococcus*, possibly affecting human phenylalanine and ABC transporter-related metabolic pathways.

The present study identified two bacteria, g_*Faecalibacterium* and g_*Agathobacter*, which might have favorable effects on PLWH. g_*Faecalibacterium* is the dominant bacterium in the human intestine, representing >5% of the total number of bacteria in the microbiota (29). g_*Faecalibacterium* is functionally very active, being one of the best butyrate producers in the gastrointestinal tract (30). g_*Faecalibacterium-*produced butyrate could influence homeostasis and physiological functions to support health. Butyrate, an SCFA, has important functions in the physiology of the gut. It affects the intestinal cell life cycle in a variety of ways and has various beneficial health effects, including pathogen invasion prevention, immune system modulation, and restraining cancer progression (11, 31, 32). Furthermore, the anaerobic, Gram-positive bacteria g_*Agathobacter* is a species of a new genus in the family *Lachnospiraceae*. This genus mainly produces lactate, hydrogen, acetate, and butyrate via fermentation (33). Levels of butyrate are decreased in patients with HIV (34, 35); thus, g_*Faecalibacterium* and g_*Agathobacter* might represent potential probiotics for these patients. In addition, there was a correlation between the marked decrease in g_*Faecalibacterium* and g_*Agathobacter* and the fecal levels of N-acetyl-L-glutamate 5-semialdehyde, 11b-hydroxyandrost-4-ene-3,17-dione, and 17alpha,21-dihydroxypregnenolone, with possible effects on human metabolic pathways, such as arginine biosynthesis and steroid hormone biosynthesis.

Additionally, g_*Enterococcus* might affect behavior and brain function via the brain-gut-microbiota axis. Despite being a member of the normal gut flora, g_*Enterococcus* (phylum *Firmicutes*) was recognized as an opportunistic pathogen. Its two main species, *Enterococcus faecalis* and *Enterococcus faecium*, cause endocarditis, urinary tract infections, and other hospital-acquired infections (36). Levodopa is the drug used for dopamine replacement therapy in Parkinson’s disease treatment regimens. Recent studies have confirmed that g_*Enterococcus* activates tyrosine decarboxylase, possibly resulting low levels of intracranial dopamine and low levels of levodopa in the nervous system (37, 38). We observed that g_*Enterococcus* and N-acetyl-L-phenylalanine were strongly and negatively correlated, which might affect the metabolism of phenylalanine. Attenuated phenylalanine conversion might cause hyperphenylalaninemia, which may lead to impaired cerebral function and neuropsychiatric disorders (11, 39). Moreover, g_*Enterococcus* correlated positively with 11b-hydroxyandrost-4-ene-3,17-dione and 17alpha,21-dihydroxypregnenolone, which might influence human steroid hormone biosynthesis.

This study revealed the interactions between the intestinal microbiome and host between PLWH and HC individuals by combined analyses of the gut microbiota and the metabolome. The health of PLWH might benefit by adopting measures including altering the gut microbiota by reducing the levels of potential pathobionts (e.g., g_*Enterococcus*), raising the levels of potential probiotics (e.g., g_*Faecalibacterium* and g_*Agathobacter*), and reducing the levels of certain metabolites (e.g., N-acetyl-L-phenylalanine and trehalose), which might be achieved by changes in their diet or the addition of nutritional supplements. However, this study had limitations. First, we did not assess other important microorganisms (i.e., fungi, bacteriophages, and viruses) which might also affect the interaction between the host and gut microorganism and might be associated with diseases. Second, the cross-sectional design meant that no conclusions on causality could be drawn. Third, the sample size of this preliminary pilot study was limited. Consequently, further experiments are required to verify these results. The primary focus of this study was the overall impact of HIV infection on the human gut microbiota and its associated metabolome. Our results provide insights into the physiological consequences HIV infection, as reflected in alterations to the gut microbiota and metabolome. Further *in vitro* and *in vivo* studies are required to verify the causal mechanisms among HIV infection, physical disorders, and the gut microbiota and metabolome.

### Conclusion

A combination of 16S rRNA gene sequencing and LC-MS untargeted metabolomics was used to reveal the interactions between the microbiome and the host in PLWH. Our results suggested that HIV/AIDS not only disturbs the gut microbiome but also leads to alterations in several human metabolic pathways.

## Data Availability

The raw microbiome data are accessible in the National Center for Biotechnology Information database with accession number PRJNA1057804. The raw metabolomics data set is available in the MetaboLights database (www.ebi.ac.uk/metabolights/studies) under study identifier MTBLS9264. All the data supporting the conclusions are provided within the article and the supplemental materials.

## SUPPLEMENTAL MATERIAL

Supplemental material spectrum.00238-24-s0001.docx Table S1; Fig. S1 and S2.

ASM does not own the copyrights to Supplemental Material that may be linked to, or accessed through, an article. The authors have granted ASM a non-exclusive, world-wide license to publish the Supplemental Material files. Please contact the corresponding author directly for reuse.
