# Supplementary material for: Analysis of the gut microbiota and fecal metabolites in people living with HIV
Source: Microbiol Spectr. 2024 Sep 18;12(11):e00238-24. doi: 10.1128/spectrum.00238-24 (PMC11537111; doi:10.1128/spectrum.00238-24)
Supplement: Supplemental material — Table S1; Fig. S1 and S2. [file spectrum.00238-24-s0001.docx]

**Supplementary Materials**

**Table S1.** Baseline characteristics of the discovery cohort.

| Characteristic | People living with HIV (n = 70) | Healthy Controls  (n = 34) | P-value |
| --- | --- | --- | --- |
| Age year (mean ± SD) | 42.48 ± 2.17 | 42.85 ± 11.59 | 0.8853 |
| Male/female (No.) | 69/1 | 34/0 | 0.4837 |


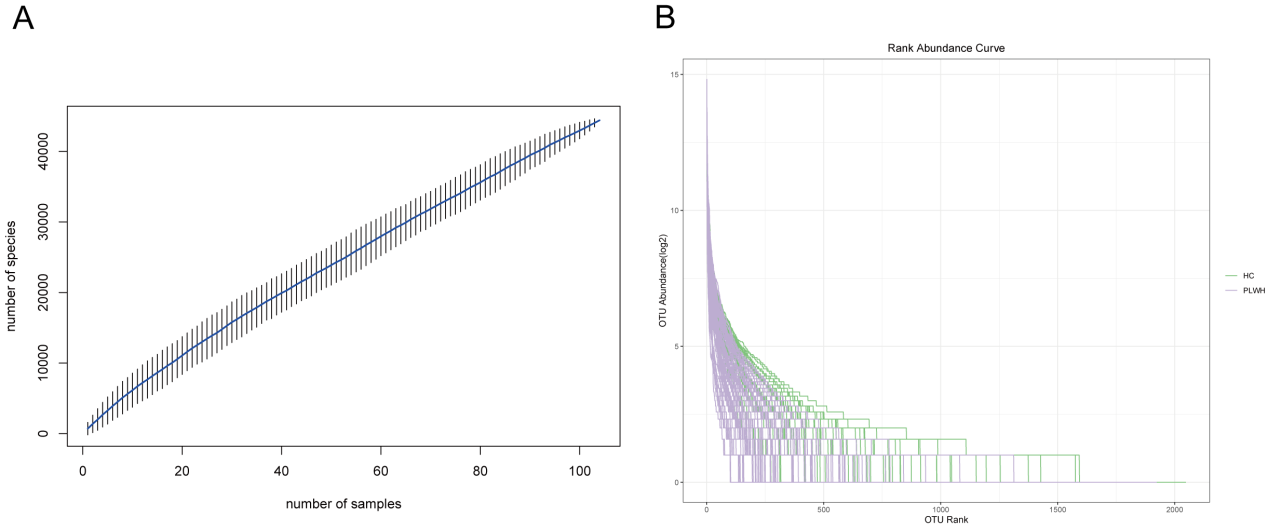


**Figure S1. 16S rRNA gene sequencing of fecal bacteria.** (A) Species accumulation curve of all sequenced samples. The horizontal axis represents the sample size, and the vertical axis represents the number of ASVs/operational taxonomic units (OTUs) detected. (B) RankAbundance curve between the HC and PLWH groups. On the horizontal axis, OTUs are sorted according to the number of sequences they contain.


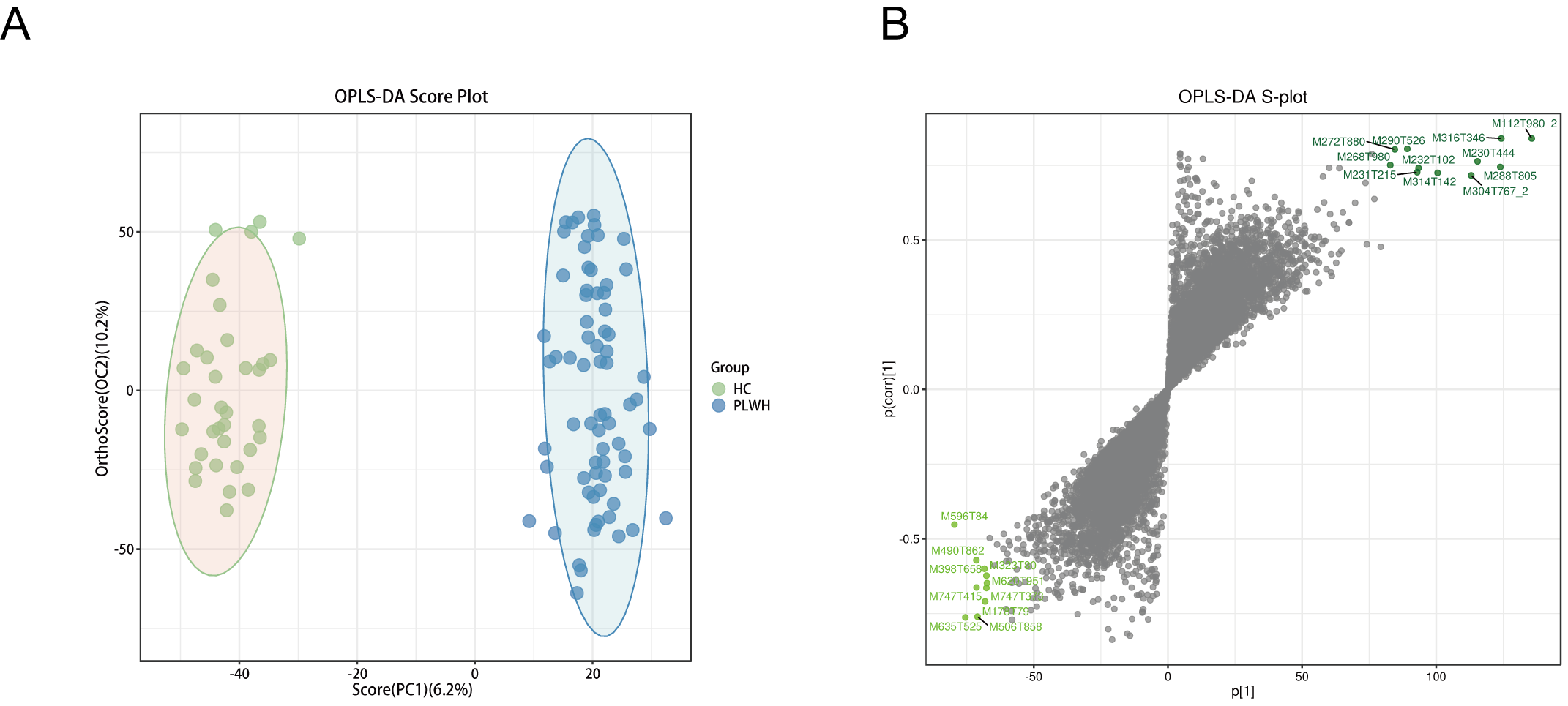


**Figure S2. Liquid chromatography-mass spectrometry analysis of fecal**

**metabolites.** (A) Orthogonal partial least squares discrimination analysis (OPLS-DA) between the HC and PLWH groups. (B) OPLS-DA S-plot. The horizontal axis represents the characteristic values of the effects of metabolites between the HC and PLWH groups, and the vertical axis represents the correlations between the sample scores and the metabolites. The closer the metabolites are to the upper right corner and the lower left corner, the greater the difference. PC1, principal component 1.
